# Applicability of risk compensation to the relationship between health behaviors and COVID-19 vaccination among inpatients in Taizhou, China

**DOI:** 10.1007/s10389-023-01865-w

**Published:** 2023-03-15

**Authors:** Liguang Chen, Tao-Hsin Tung, Xinxin Zhang, Gang Wang

**Affiliations:** Taizhou Hospital of Zhejiang Province affiliated to Wenzhou Medical University, Taizhou, 317000 China

**Keywords:** COVID-19, COVID-19 vaccine, Risk compensation, Health behaviors

## Abstract

**Aim:**

Based on the risk compensation theory, this study was designed to investigate the relationship between health behaviors of inpatients and COVID-19 vaccination during the epidemic with regard to the Omicron variant of the severe acute respiratory syndrome coronavirus 2 (SARS-CoV-2) in Taizhou, China.

**Subject and methods:**

We conducted an online self-administered survey with a group of inpatients in a grade III, class A hospital in Taizhou, China, from February 27, 2022, to March 8, 2022. A total of 562 complete questionnaires were collected, and 18 questionnaires completed in under 180 seconds were rejected, leaving a total of 544 (96.8%) valid questionnaires collected. The participants who had received a COVID-19 vaccine were asked to describe the differences in their health behaviors before and after the vaccination, and the data were analyzed using SPSS Statistics version 22.0 software.

**Results:**

There were significant differences in the percentage of individuals wearing masks (97.2% and 78.9%, *P* < 0.001) and the percentage of hand washing after taking off the mask (89.1% and 63.2%, *P* < 0.001) between the inoculated group and the uninoculated group; however, there were no significant differences in other health behaviors. The participants showed better health behaviors (handwashing and wearing a mask) after the vaccination than prior to it.

**Conclusions:**

Our findings suggest that the Peltzman effect did not increase risk behaviors during the Omicron epidemic. There was no reduction in health behaviors among inpatients after the COVID-19 vaccine, which may have even improved their health behaviors.

## Introduction

The novel coronavirus disease 19 (COVID-19) pneumonia, caused by the severe acute respiratory syndrome coronavirus 2 (SARS-CoV-2), has infected 490 million people worldwide and has caused over 6.16 million deaths (World Health Organization 2021). Vaccines have proven to be an extremely effective approach to combat prior epidemics (Wadman and You 2017). A global survey showed that respondents were willing to receive the COVID-19 vaccine (Zhang et al. 2021). With the emergence of a new Omicron mutant strain, the prevention and control of the epidemic have become more complicated and arduous. There were as many as 32–50 variants of Omicron, and 30 mutation points were concentrated on the spike proteins, which were closely related to pathogenicity and antigenicity. As a result, experts are generally concerned that the strain is more transmissible than the Delta variant, with the potential for more secondary infections and higher “drug resistance” to the vaccine. Wearing masks and gloves and handwashing in public places are some of the key measures to prevent transmission and to control the spread of the COVID-19 pandemic (Ogoina 2020; Przekwas and Chen 2020). Therefore, during the Arcanum epidemic, compliance with health behaviors was important in reducing the risk of individual infections and controlling the spread of the epidemic.

Risk compensation indicates that when people are perceived to be at risk, they naturally adjust their behaviors to compensate and reduce the risk (Hedlund 2000). However, as the novelty of the threat wears off, the effect of risk compensation tends to fade over time (Hedlund 2000). This phenomenon, in which individuals respond to safety measures with a compensatory increase in risk behaviors, is known as the "Peltzman effect,” named after the University of Chicago economist Sam Peltzman (Hedlund 2000; Peltzman 1975). During the decades since its initial description, the presence of the Peltzman phenomenon has been inconsistent, since it has been observed with some security interventions but not with others (Hedlund 2000). There is also a direct relationship between risk compensation mechanisms and behavioral differences before and after vaccination. Therefore, during the epidemic with the Omicron variant, we conducted a questionnaire survey on this issue and assessed risk compensation between health behaviors of hospitalized patients in Taizhou and COVID-19 vaccination.

## Materials and methods

### Research design and population

**Fig. 1:**
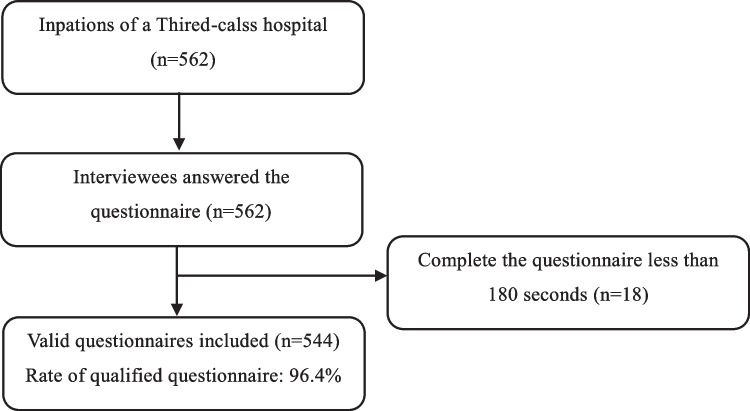
Flowchart of the method for sample size determination

We conducted an anonymous, online, cross-sectional survey through WeChat, China's largest online survey platform, which targeted hospitalized patients in Taizhou, China. As shown in Fig. 1, a total of 562 people were asked to report their health behaviors via WeChat between February 27, 2022, and March 8, 2022. A total of 562 completed questionnaires were collected, among which there were 544 (96.8%) valid questionnaires and 18 questionnaires that took less than 180 seconds to complete. We examined participants' gender, knowledge, employment categories, and health behaviors such as wearing a mask, hand washing, and gatherings. The patients’ experiences of negative emotions (anxiety, panic, and fear) were also recorded. Participants who had received the COVID-19 vaccine were asked to describe the differences between their health behaviors before and after the vaccination. This investigation was exempt from the need for informed consent and was approved by the Ethics Committee of Enze Hospital, Zhejiang Province, China. The participants were anonymous. The COVID-19 vaccine in this study was a Inactivated vaccine produced by Sinovac. The Chinese government has provided the COVID-19 vaccine to the public free of charge.

### Questionnaire

Our questionnaire was based on a survey conducted by Sun et al. (2022) on the protective health behaviors of hospital staff during the COVID-19 pandemic in 2019. The questionnaire included the following relevant items.

1. Basic demographic information, including gender, educational level, and professional/technical titles.
2. Consequences of health behaviors during the Omicron epidemic: “What about wearing masks and washing hands?” (Always, Usually, Seldom, Never)
3. The status of COVID-19 vaccination, assessed by the questions: “Did you get the COVID-19 vaccine?” and “If you did, when did you get it?”
4. Comparing the health behaviors of wearing a mask, hand washing, and gatherings before and after the inoculation of COVID-19 vaccine during the epidemic with the Omicron variant.
5. Experience of negative emotions (anxiety, panic, or worry) as a result of the prevalence of the Omicron variant.

### Statistical analysis

All data were analyzed with IBM SPSS Statistics version 22.0. In univariate analysis, the *χ*^2^ test was used to assess the categorical variables between fatigued and non-fatigued participants. The main results of this investigation were the health behavior during the Omicron mutant epidemic and the difference in health behaviors before and after the inoculation. All participants provided answers about the health behaviors they followed, and whether they had been vaccinated. Follow-up questions, such as the time of the first dose, were answered by the vaccinated participants. A value of *P* < 0.05 was considered to represent a statistically significant difference between the test groups.

## Results

### Characteristics of the study population

A total of 544 participants completed the survey. The sample consisted of 191 men (35.1%) and 353 women (64.9%) who reported health behaviors and negative emotions following an outbreak of COVID-19. Among all hospitalized patients, 506 (90.0%) received at least one dose of the COVID-19 vaccine, while 38 did not receive the vaccine. The demographic variables age, education, and occupation were also recorded. The proportions of females, middle-aged persons, and highly educated individuals in the COVID-19 vaccine group were 66.6% (*P* = 0.004), 55.7% (*P* < 0.001), and 53.6% (*P* = 0.011), respectively. The proportion of farmers in the uninoculated group was 36.8% (*P* = 0.009). These results are shown in Table 1.

**Table 1:** Baseline characteristics of the participants

	Variables	All *n* = 544(%)	Vaccination *n* = 506(%)	No vaccination *n* = 38(%)	*χ*^2^	*P*
Sex	Men	191 (35.1)	169 (33.4)	22 (57.9)	9.309	0.004
	Women	353 (64.9)	337 (66.6)	16 (42.1)		
Age (years)	< 30	146 (26.8)	142 (28.1)	4 (10.5)	20.638	< 0.001
	≥ 30 and < 60	299 (55.0)	282 (55.7)	17 (44.7)		
	≥ 60	99 (18.2)	82 (16.2)	17 (44.7)		
Education	Junior high and below	261 (48.0)	235 (46.4)	26 (68.4)	6.841	0.011
	High school and above	283 (52.0)	271 (53.6)	12 (31.6)		
Position	Workers or professionals	117 (21.5)	111 (21.9)	6 (15.8)	13.583	0.009
	Business units	87 (16.0)	83 (16.4)	4 (10.5)		
	Housewives	73 (13.4)	68 (13.4)	5 (13.2)		
	Farmers	87 (16.0)	73 (14.4)	14 (36.8)		
	Other	180 (33.1)	171 (33.8)	9 (23.7)		

### Inpatients’ health behaviors during the epidemic with the Omicron variant

In our survey, we examined different health behaviors of the vaccinated and unvaccinated respondents. Table 2 shows that the wearing of masks and handwashing after removing masks were associated with vaccination, while other health behaviors showed no association.

**Table 2:** Behaviors of healthcare workers during the COVID-19 pandemic

	Variables	All *n* = 544 (%)	Vaccination *n* = 506 (%)	No vaccination *n* = 38 (%)	*χ*^2^	*P*
Negative emotions due to the epidemic	No Yes	435 (80.0) 109 (20.0)	401 (79.2) 105 (20.8)	34 (89.5) 4 (10.5)	2.306	0.146
Mask wearing	No Yes	22 (4.0) 522 (96.0)	14 (2.8) 492 (97.2)	8 (21.1) 30 (78.9)	25.926	**< 0.001**
Type of masks	N95/Normal masks Surgical mask	104 (19.1) 440 (80.9)	94 (18.6) 412 (81.4)	10 (26.3) 28 (73.7)	1.369	0.283
Time of changing masks	< 6 h 6–12 h > 12 h	256 (47.1) 132 (24.3) 156 (28.7)	242 (47.8) 121 (23.9) 143 (28.3)	14 (36.8) 11 (28.9) 13 (34.2)	1.712	0.425
Whether the nose tip is not pinched when wearing a mask	No Yes	246 (45.2) 298 (54.8)	229 (45.3) 277 (54.7)	17 (44.7) 21 (55.3)	0.004	1.000
Handwashing after taking off masks	No Yes	69 (12.7) 475 (87.3)	55 (10.9) 451 (89.1)	14 (36.8) 24 (63.2)	19.247	**< 0.001**
Attending a gathering of 10 or more people	No Yes	351 (64.5) 193 (35.5)	323 (63.8) 183 (36.2)	28 (73.7) 10 (26.3)	1.498	0.291

### Differences in health behaviors among vaccinated individuals

**Fig. 2:**
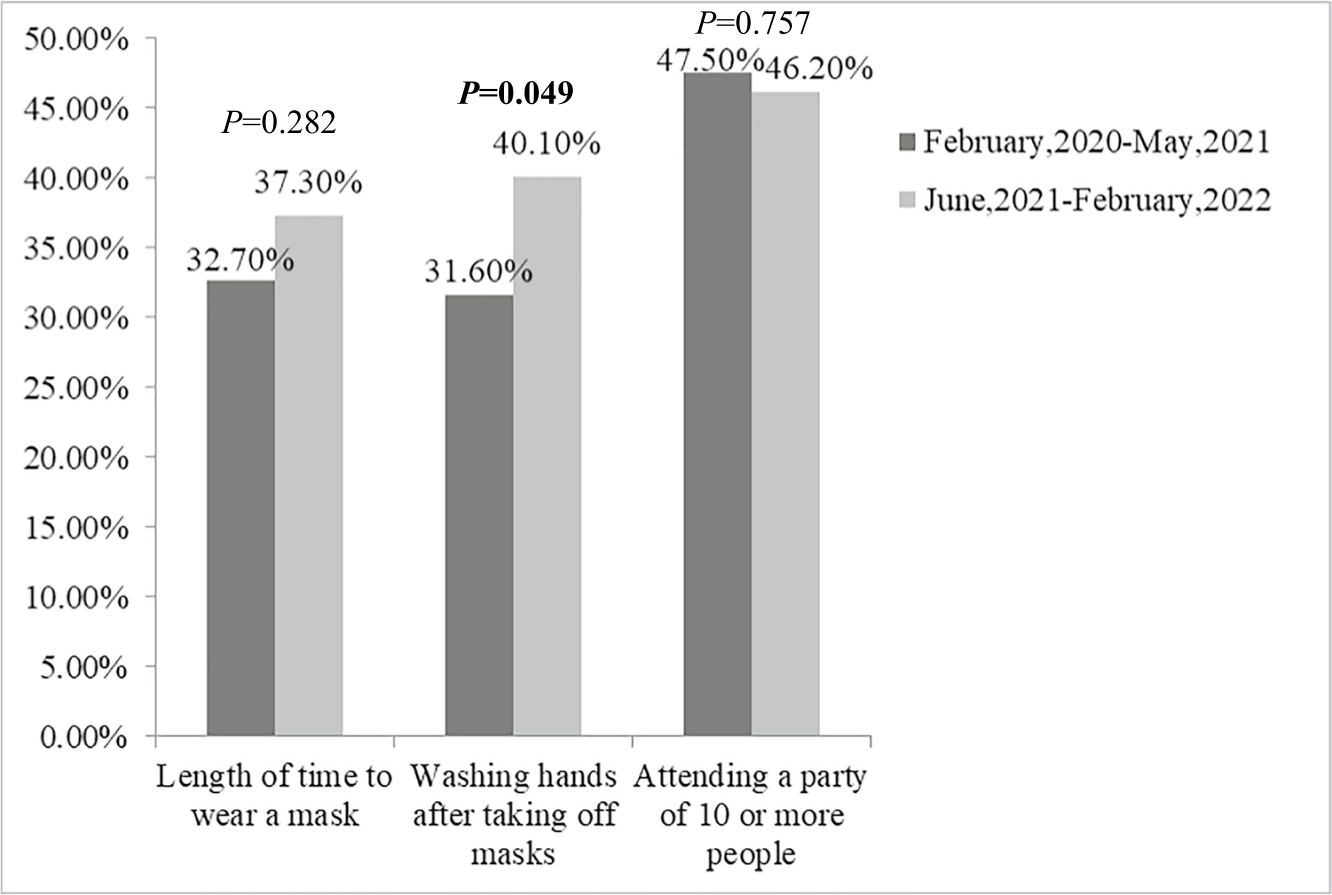
Association between the time of the first vaccine and increased health behaviors

**Fig. 3:**
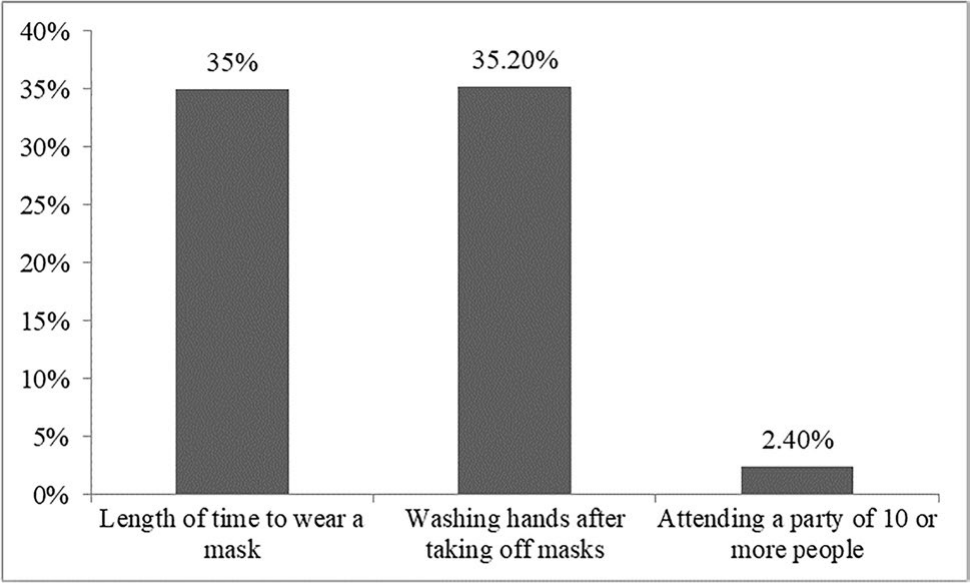
Increased health behaviors post-inoculation compared to pre-inoculation among recipients of the COVID-19 vaccine

As shown in Fig. 2, there was a significant difference in the number of times handwashing was done after vaccination (*P* = 0.049), depending on the time of the first vaccination. By comparing health behaviors before and after the vaccination, we found significant increases in mask-wearing duration, handwashing frequency, and participation in gatherings of more than 10 people in our sample (Fig. 3).

### Negative emotions (anxiety, panic, and fear)

During the epidemic with the Omicron variant, our survey data showed that 79.96% of the participants had never experienced negative emotions, while only 4.76% of the participants experienced an increase in negative emotions after receiving the COVID-19 vaccine. These results are shown in Table 3.

**Table 3:** Experience of negative emotions (anxiety, panic, and fear) related to COVID-19 prevalence and vaccine inoculation

Part 1. Experience of negative emotions during COVID-19 prevalence	
Groups	*n* = 544 (%)
Always	4 (0.74)
Usually	9 (1.65)
Seldom	96 (17.65)
Never	435 (79.96)
Part 2. Experience of negative emotions post-inoculation compared to pre-inoculation among recipients of the COVID-19 vaccine	
Groups	*n* = 105 (%)
Increased	5 (4.76)
Equal	48 (45.72)
Decreased	52 (49.52)

## Discussion

The study showed that inpatients who received the COVID-19 vaccine did not necessarily have fewer nondrug interventions, such as hand-washing and wearing masks than unvaccinated inpatients. Furthermore, our research showed that people who get vaccinated early have better health behaviors and perform better after vaccination. The rate of wearing a face mask was 97.2% (*P* < 0.001), and the rate of handwashing was 89.1% (*P* < 0.001).

Compared with the investigation by Sun et al. (2022), the rate of increased health behaviors of hospitalized patients after vaccination in this study was high. Compared with pre-inoculation, the time wearing a mask increased by 35%, and the frequency of handwashing increased by 35.2%, whereas in the study by Sun et al. the time wearing a mask increased by 17% and the frequency of washing hands increased by 18%. Sun et al. conducted a questionnaire survey from May 18 to 21, 2021, when the domestic epidemic was relatively stable, and there was no large-scale outbreak. The questionnaire in this study was conducted from February 27, 2022, to March 8, 2022, during the epidemic with the Omicron variant in Hong Kong and the Jilin province. People’s health behaviors may have been affected by the epidemic at that time, leading to an increase in health behaviors. From December 31, 2021, to March 23, 2022, the fifth round of COVID-19 in Hong Kong killed 6356 people. The main strain during the fifth round of the epidemic in Hong Kong was Omicron BA.2, which was the most infectious but the least pathogenic of all SARS-CoV-2 strains. If K417, NE484A, or K501Y mutations are present in the spike proteins of novel coronaviruses (Li et al. 2021), the immune escape ability of the strains can be enhanced. The variant also contained other important amino acid mutations that alter the virus's sensitivity to neutralizing antibodies, leading the virus to evade the neutralizing effects of anti-infectious antibodies caused by previous COVID-19 infections or vaccinations, leading to a massive breakthrough infection (Garcia-Beltran et al. 2021; Greaney et al. 2021).

The main sources of information mentioned in studies, such as that by Bhagavathula (2022), are the news media and government websites. Approximately one third of the participants reported that they regularly consumed news media (television/video, newspapers, radio, and magazines), whereas a quarter (24.3%) relied on the websites of national and international health agencies, including the World Health Organization （WHO）, Centers for Disease Control (CDC) and Prevention, and Ministry of Health （MOH）, for Omicron information. In addition, 39% of the participants sometimes discussed the Omicron strain with family and friends. The government has issued a variety of guidance documents to advise the public through WeChat, radio, news, and other means of the daily real-time release of relevant data. Simultaneously, all-level units and factories also set up epidemic prevention staff to guide and supervise the health behavior of employees within the department.

The participants in this study were inpatients. In public places, such as hospitals, special personnel has been arranged at the entrance to carry out health code, stroke code, and personnel flow track inquiries, and patients and their families are strongly advised to wear face masks. For those who do not wear masks, there is staff to remind them or issue masks to enhance the mask-wearing behavior. These measures allow people who have received the new vaccine to remain vigilant about wearing masks and to have access to medical surgical masks in public places. Even if they have forgotten their face masks, the compliance with health behaviors among inpatients increases.

These measures are effective and suitable for the national conditions of the “dynamic zero” strategy. The government uses nondrug interventions (NPI) to prevent serious outbreaks caused by the Omicron variant. First, this includes limiting the large-scale movement of people and increasing inter-provincial and inter-city traffic restrictions to reduce the cross-regional transmission of the new coronavirus. Second, the early detection and isolation of newly coronavirus-positive people and tracking of close contacts and large-scale personnel using nucleic acid screening have been emphasized. Third, the measures include increasing interpersonal distance; canceling or postponing large-scale activities, gatherings, and close-distance business entertainment places; delaying the resumption of business and school; and maintaining good personal hygiene habits. These characteristics may make inpatients more dependent on NPI and influence the outcome of health behaviors.

Although the protective effect of the new COVID-19 vaccine is declining, the protective effect on severe cases and deaths remains. However, antibodies further increase after the third injection of a “reinforcing needle”; thus, the policy of “should hit, must hit” should still be carried out. The results of the questionnaire showed that the unvaccinated groups included mainly male individuals (57.9%, *P* = 0.004), individuals with low education (68.4%, *P* = 0.011), people employed in agriculture (36.8%, *P* = 0.009), and the elderly (44.7%, *P* < 0.001). As the world is experiencing a wave of infections with the Omicron mutation, inevitably spreading to every continent, there is an urgent need for greater efforts through educational interventions targeting the general population, to improve individuals’ knowledge and understanding of the Omicron variant and to promote healthy behaviors. In particular, we need to strengthen education efforts for men, the less educated, the elderly, and farmers and to develop relevant and feasible measures to guide people to actively vaccinate. During an outbreak, it is especially important to strengthen the health of this group of people and to promote their health behaviors. However, one should not expect two or even three shots to be safe. Instead, the same strict precautions should be maintained.

## Limitations

There are several limitations to this study. This was a cross-sectional study conducted between late February and early March 2022, when an alarming number of cases of infection with the Omicron variant were reported worldwide that could affect the general health behavior of the population. The data presented in this study were self-reported and depend on participants’ honesty and recall ability, so they may be affected by recall bias. This study was limited to inpatients in a grade III, class A hospital in Taizhou. In the future, the data will be included in a larger multicenter sample to provide evidence for a better response to the COVID-19 epidemic. In addition, factors such as internet access, time changes, and language barriers may influence participation. Despite these limitations, the results of this study provide insight into changes in the health behaviors of hospitalized patients at the peak of the Omicron mutant pandemic.

## Conclusions

In this study, the concept of risk compensation was not applicable to our results. After being vaccinated with a COVID-19 vaccine, the health behaviors of the population did not decrease and may have even improved. Further large-scale population surveys are needed to determine the applicability of the risk compensation theory in association with health behaviors and COVID-19 vaccination.

## Data Availability

Not applicable.
